# Asymptomatic and Persistent Elevation of Pancreatic Enzymes in an Ulcerative Colitis Patient

**DOI:** 10.1155/2013/415619

**Published:** 2013-05-14

**Authors:** Elisa Liverani, Filippo Leonardi, Lucia Castellani, Carla Cardamone, Andrea Belluzzi

**Affiliations:** Department of Internal Medicine and Gastroenterology, Sant'Orsola-Malpighi Hospital, University of Bologna, Via Massarenti 9, 40138 Bologna, Italy

## Abstract

Azathioprine has been extensively used in the management of inflammatory bowel diseases. It might cause pancreatic damage in the form of either asymptomatic elevation in serum amylase/lipase or overt acute pancreatitis. Here we report the case of a 61-year-old patient with ulcerative colitis who had been treated with azathioprine for three years, achieving clinical remission. During treatment he presented an asymptomatic elevation of serum pancreatic enzymes, without any signs of pancreatitis at imaging. This evidence brought us to reassess the drug dosage, without achieving a normalization of biochemical analysis. Autoimmune pancreatitis was excluded. One year after the suspension of azathioprine, we still face persistent high levels of amylase/lipase. Normalization of enzymatic values in patients who develop intolerance to azathioprine, in the form of either asymptomatic elevation in serum amylase/lipase or overt acute pancreatitis, is usually achieved in about two months after stopping drug intake. Asymptomatic elevation in serum pancreatic enzymes in the absence of pancreatic disease is reported in the literature and defined as “Gullo's syndrome,” but nobody of the subjects studied had been treated in the past with pancreatotoxic drugs. Might this case be defined as “benign pancreatic hyperenzymemia”?

## 1. Introduction

Azathioprine (AZA) has been extensively used in the management of inflammatory bowel diseases (IBD). It plays an important role as steroid sparing agent in inducing and maintaining a stable remission of disease. This immunosuppressive medication has been associated with a number of side effects, including bone marrow suppression, liver injury, acute pancreatitis, and others. The etiopathogenesis of AZA-induced pancreatic damage, in the form of either asymptomatic elevation in serum amylase/lipase or overt acute pancreatitis, is still not clear, but some authors propose that a possible immune-mediated mechanism or a hypersensitivity to the drug could be involved. Patients treated with AZA have to check routinely biochemical tests to exclude the potential pancreatotoxicity. 

Hyperamylasemia and hyperlipasemia in IBD patient never treated with pancreatotoxic drug are reported in the literature. Several authors suggest a latent involvement of the pancreas as an extraintestinal manifestation of IBD, and some others lean towards an abnormal reabsorption of amylase/lipase due to the increased permeability of the inflamed mucosa. In this report, we also review the literature concerning nonspecific elevations of serum pancreatic enzymes in IBD patients.

## 2. Case Presentation

The patient is a 61-year-old man, presenting a history of ulcerative colitis (UC) known since April 2007. He came to our attention in March 2008, for severe exacerbation of the intestinal disease, characterized by abdominal pain, diarrhea with blood and mucus in stool, tenesmus, and fever. Laboratory tests showed an elevation of inflammation indexes (ESR, CRP, and *α*_1_-acid glycoprotein) with leukocytosis, while liver function tests and pancreatic enzymes were essentially normal (normal range: 30–100 U/L for serum amylase; <60 U/L for serum lipase).

**Figure 1:**
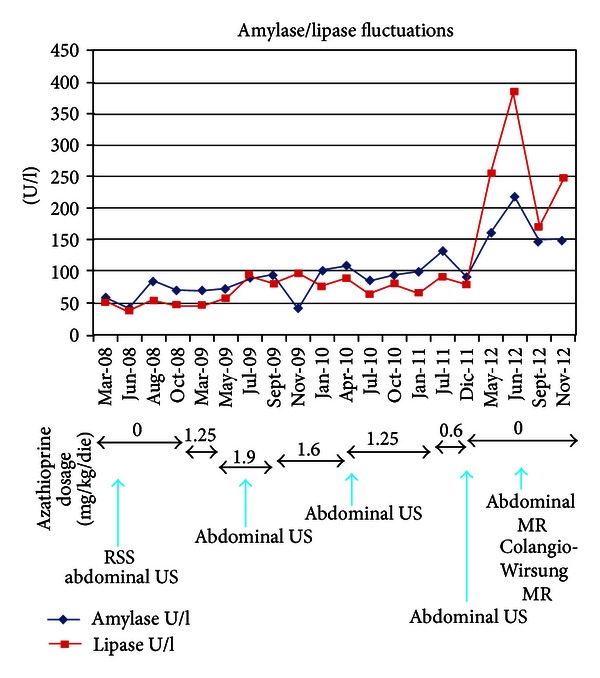
Amylase and lipase fluctuations. The graphic shows amylase and lipase fluctuations from March 2008 to November 2012, in correspondence with therapy modifications: AZA dosage relating to each period is reported. The blue arrows show when instrumental investigations were performed. RSS: rectosigmoidoscopy; US: ultrasonography; MR: magnetic resonance.

**Figure 2:**
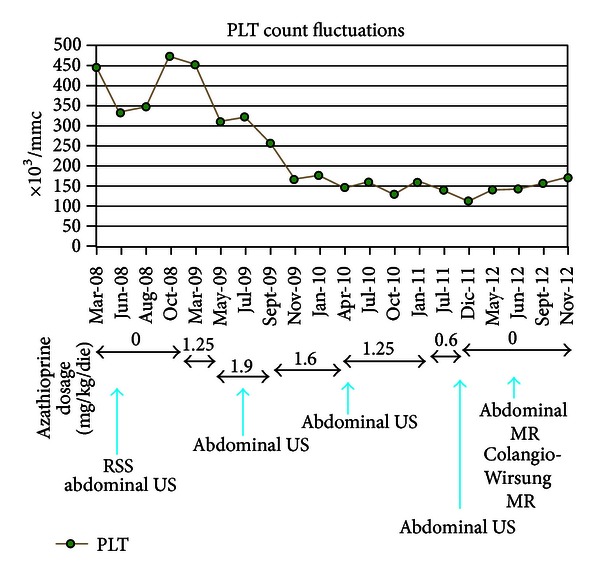
Platelets count fluctuations. The graphic shows platelets fluctuations from March 2008 to November 2012, in correspondence with therapy modifications: AZA dosage relating to each period is reported. The blue arrows show when instrumental investigations were performed. PLTs: platelets count; RSS: rectosigmoidoscopy; US: ultrasonography; MR: magnetic resonance.

We performed a rectosigmoidoscopy during the hospitalization, that showed an ulcerative proctosigmoiditis of severe activity, complicated by a pseudomembranous colitis. This finding was confirmed by the presence of clostridium difficile toxin both on stool samples and on bioptical specimens. The patient was treated with vancomycin (as antibiotic therapy of pseudomembranous colitis) and with an association of mesalamine and steroids (as antiinflammatory therapy for UC). This association therapy achieved a clinical improvement, but after steroid decalage the patient faced an immediate relapse of the intestinal disease. He was hospitalized again in June 2008 and treated with high doses of endovenous corticosteroid obtaining a clinical improvement. He still presented normal biochemical values, in particular he had normal serum amylase and lipase levels (Figures 1 and 2).

After discharge he was followed up by our specialized IBD outpatients' department.

In the following months, during steroid decalage, the patient experienced mild relapse of the disease, so we decided to introduce AZA 100 mg/die (1.25 mg/kg/die) in January 2009. The patient performed controls of biochemical analysis every three months. In March 2009, the clinical state improved, but the patient still complained abdominal discomfort and occasional diarrhea, so we decided to increase the AZA dosage to 150 mg/die (1.9 mg/kg/die). Nine months after starting AZA therapy, in September 2009, UC remission was achieved, but the biochemical analysis showed a minimal hyperlipasemia (94 U/L), while amylase levels persisted normal. For this reason, we decided to decrease the AZA dosage to 125 mg/die (1.6 mg/kg/die) without obtaining normalization of lipase; in actual fact, one year after starting AZA therapy, we faced a further elevation of both serum lipase and amylase levels (resp., 76 U/L and 102 U/L in January 2010). In this hematological control we also noticed a decrease in platelets count (166.000/mm^3^; normal range: 150.000–450.000/mm^3^), not ascribable to splenic sequestration (no evidence of splenomegaly at ultrasonography). In April 2010 the patient persisted asymptomatic, but hyperamylasemia, hyperlipasemia, and thrombocytopenia were still present (resp., 109 U/L, 88 U/L, and 145.000/mm^3^). Therefore, the drug dosage was decreased again to 100 mg/die (1.25 mg/kg/die). Levels of pancreatic enzymes at biochemical analysis kept fluctuating over the upper normal values in the following year. In July 2011, we decided to decrease AZA dosage to 50 mg/die (0.6 mg/kg/die), because of a further increase of amylase/lipase (resp., 132 U/L and 90 U/L), associated with a worsening thrombocytopenia (138.000/mm^3^). No improvement in biochemical analysis and in platelets count was obtained in the following six-month period (Figures 1 and 2). Finally in December 2011, after three years of AZA intake, we agreed with the patient to stop the current therapies, persisting the disease on stable remission.

The suspension of AZA did not cause a worsening of clinical conditions linked to UC, but in January 2012 the patient complained overnight itching on the legs. This symptom was studied through haematological consultation, which excluded any kind of haematological disorders.

Six months after the AZA suspension, pancreatic enzymes unexpectedly showed a further increase (serum amylase: 161 U/L, lipase: 255 U/L in May 2012). (Figure 1) In order to rule out autoimmune pancreatitis (AIP), we measured IgG levels, particularly the dosage of IgG4 subclass, that resulted within normal values. Antinuclear antibodies (ANA) were absent, as well as the titers of the other NOS autoantibodies. We also evaluated the response to steroids, performing a diagnostic trial of two weeks with prednisolone, without obtaining a biochemical normalization of pancreatic enzymes (resp., 218 U/L, 386 U/L in June 2012). (Figure 1) Imaging features of the pancreatic parenchyma and pancreatic duct did not meet diagnostic criteria for AIP. As regards instrumental investigations, the patient made an US follow up during these years, which never provided evidence of pathological involvement of abdominal organs. The MR of the abdomen and the Colangio-Wirsung MR did not show any alteration in the size, course, and morphology of extrahepatic and intrahepatic bile ducts, neither in the pancreatic ducts nor in parenchyma [1, 2].

At the present moment, one year after the suspension of AZA, we still face hyperamylasemia and hyperlipasemia (149 U/L; 247 U/L, resp.), while urinary amylase is absent.

Our patient has no risk factors predisposing to pancreatitis (no evidence of gallstones, no alcohol consumption, no hypertriglyceridemia, and no hypercalcemia) and has never complained symptoms typical of flogistic involvement of the pancreatic gland. Red blood cells and total leukocytes counts, hemoglobin, and inflammation indexes (ESR, CRP, and *α*_1_-acid glycoprotein) have always resulted normal since the achievement of UC remission in September 2009. Furthermore, we have never detected an elevation of liver function enzymes or alteration in cholestasis index or renal function (levels of serum creatinine, urea, and electrolytes have always been within the normal range). We also point out the negativity of autoantibodies typical of autoimmune cholestatic liver diseases.

## 3. Discussion

An elevation of serum amylase and lipase without symptoms of pancreatitis is documented to be more frequent in IBD patients than in controls [3–5]. Various possible explanations for asymptomatic hyperamylasemia and hyperlipasemia in IBD patients have been proposed and are still matter of discussion.

Several authors suggest a latent involvement of the pancreas as an extraintestinal manifestation of the inflammatory bowel disease. The demonstration of autoantibodies against the exocrine pancreas, causing a direct pancreatic damage with enzyme leakage into the bloodstream, and the histological proof of a granulomatous inflammation of the pancreas may support this view.

Another possible explanation is linked to the abnormal reabsorption of pancreatic amylase/lipase from the gut lumen to the bloodstream due to the increased permeability of the inflamed mucosa. In conjunction, the pancreatic enzymes elevation observed in IBD patient may be associated with the extrapancreatic lipase/amylase activity in the small bowel and in the colon; lipase and amylase may be absorbed excessively in the blood in case of an increased inflammatory activity. The latter hypothesis is supported by the results of cross-sectional study by Heikius et al. who demonstrated a correlation between the lipase increase and the histological activity and extension of the disease. In this study, patients with more extensive disease had higher pancreatic enzymes activity than those with distal involvement (proctosigmoiditis) [5]. It is important to highlight that the mentioned studies were conducted in subjects never treated with immunosuppressive therapies (neither AZA nor biological drugs) or any kind of pancreatotoxic agents. 

In our case, the UC patient has persistent pancreatic hyperenzymemia one year after the suspension of AZA, without any clinical symptoms or imaging appearance of pathological involvement of the pancreas. Platelets count is still low but within the normal range. No alterations of amylase/lipase levels and platelets count were present before starting AZA therapy. The proctosigmoiditis has always been in remission both during and after the suspension of AZA.

AZA therapy seems to be responsible of thrombocytopenia, but the same cannot be told about pancreatic hyperenzymemia. As a matter of fact, platelets count is rising, but one year after AZA suspension, levels of amylase/lipase persist elevated and are also higher than the ones reported during treatment. Patients who developed intolerance to AZA, such as asymptomatic elevation in serum amylase/lipase or acute pancreatitis, usually normalized enzymatic values in two months after suspension of AZA.

It has been documented that mesalamine (5-ASA) and related medications can also cause an elevation of serum pancreatic enzymes and/or a decrease in platelets count. Our UC patient was treated with 5-ASA (both oral administration and enemas), but neither an improvement in serum amylase/lipase levels nor in platelets count occurred after the suspension of this drug [6].

An elevation in serum pancreatic enzymes in the absence of pancreatic disease is reported in the literature and defined as “benign pancreatic hyperenzymemia” or “Gullo's syndrome” [7]. This condition can occur sporadically or in a familiar form, it is asymptomatic, and it is generally discovered incidentally. Nobody of the subjects included in this definition had been treated in the past with pancreatotoxic drugs. These authors sustain that at least one year must pass after the initial finding of pancreatic hyperenzymemia before it can be considered benign. Furthermore, they emphasize that a proper diagnosis of this condition is important because it allows the clinician to reassure the subject that this alteration is benign and does not require any kind of therapy. Nevertheless the possibility that these individuals could have an increased risk of pancreatic cancer cannot be excluded. 

To our knowledge, no similar cases have been described in the literature till now. 

Might this case be defined as “Gullo's syndrome”?
